# The lateral growth and coalesence of magma systems

**DOI:** 10.1098/rsta.2018.0005

**Published:** 2019-01-07

**Authors:** Juliet Biggs, Catherine Annen

**Affiliations:** 1 School of Earth Sciences, University of Bristol, Queen's Road, Bristol, BS8 1RJ, UK; 2 Univ. Grenoble Alpes, Univ. Savoie Mont Blanc, CNRS, IRD, IFSTTAR, ISTerre, 38000 Grenoble, France

**Keywords:** thermal models, surface deformation, transcrustal magmatic systems

## Abstract

Thermal and mechanical models of magma reservoir growth need to be reconciled with deformation patterns and structural relationships observed at active magma systems. Geophysical observations provide a series of short time-scale snap-shots (10^0^–10^2^ years) of the long-term growth of magmatic bodies (10^3^–10^6^ years). In this paper, we first review evidence for the growth of magmatic systems along structural features and the associated deformation patterns. We then define three distinct growth stages, (1) aligned melt pockets, (2) coalesced reservoirs, (3) highly evolved systems, which can be distinguished using short-term surface observations. We use two-dimensional thermal models to provide first-order constraints on the time scales and conditions associated with coalescence of individual magma bodies into large-scale reservoirs. We find that closely spaced intrusions (less than 1 km apart) can develop combined viscoelastic shells over time scales of 10s kyr and form laterally extensive mush systems over time scales of 10–100 kyr. The highest temperatures and melt fractions occur during a period of thermal relaxation after melt injection has ceased, suggesting that caldera-forming eruptions may preferentially occur long after the main intrusive activity. The coalescence of eruptible melt-rich chambers only occurs for the highest melt supply rates and deepest systems. Thus, these models indicate that, in most cases, conductive heat transfer alone is not sufficient for a full coalescence of magma chambers and that other processes involving mechanical ruptures and mush mobilization are necessary; individual melt lenses can remain isolated for long periods within growing mush systems, and will only mix during eruption or other catastrophic events. The long-term history of the magmatic system is therefore critical in determining rheological structure and hence short-term behaviour. This framework for the development of magmatic systems in the continental crust provides a mechanical basis for the interpretation of unrest at the world's largest volcanoes.

This article is part of the Theo Murphy meeting issue ‘Magma reservoir architecture and dynamics'.

## 1. Introduction

### (a) Long-term growth of magma bodies

Melt is generated in the lower crust or upper mantle, and transported through the mid-crust along structurally controlled pathways, typically ductile shear zones [1,2]. Magma which rises in discrete, short-lived pulses, stalls at rheological or lithological boundaries in the upper crust [3,4], where it cools and crystallizes forming composite bodies (after [5–7]). In this paper, we adopt the terminology of Bachmann & Bergantz [8] and use the term ‘magma reservoir' to describe a region of partially molten rock with varying proportions of crystal, melt and gas. Localized pockets in which the melt fraction is high enough (greater than 50%) to suspend crystals can be thought of as ‘magma chambers', which are sufficiently mobile to erupt. At low melt fractions (less than 50%), the crystals are in contact forming a rigid or semi-rigid framework with interstitial melt and are referred to as ‘mushes'. The orders of magnitude difference in viscosity between these components creates inherent instabilities: melt segregation likely occurs over time scales of 10^3^–10^5^ years, whereas instability, re-organization and amalgamation of the high-melt, low-viscosity components occur rapidly in months or years [9–12]. High temperatures within the reservoir alter the rheological and mineralogical properties of the surrounding rock, forming a viscoelastic contact aureole and in some cases partial melting.

**Figure 1.**
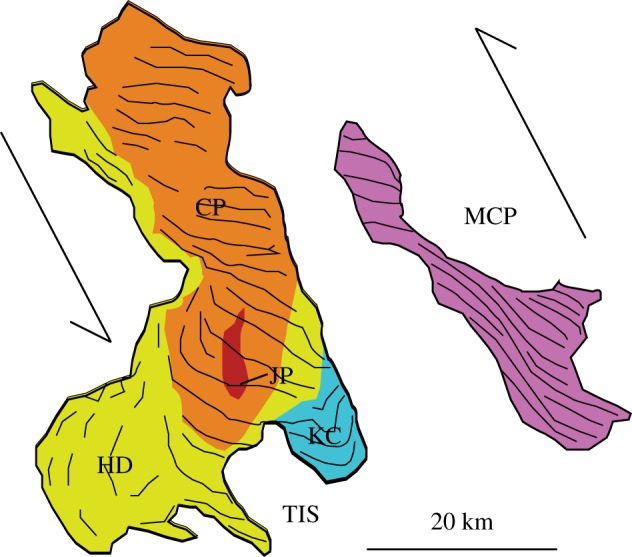
Contrasting internal structure of two NNW-SSE elongated plutons within the Sierra Nevada Batholith. The Tuolumne Intrusive Suite (TIS) is a nested set of elongated plutons (JP, HD, CP and KC), while the Mono Creek Pluton (MCP) is sheared, but internally homogeneous (adapted from [19]). (Online version in colour.)

Having undergone multiple phases of deformation, the continental crust is typically heterogeneous, with faults and lithological boundaries forming sharp rheological contrasts in a range of orientations. Plutons, which are the fully crystalline remnants of magma reservoirs, are often located on major pre-existing faults, and elongated along them [13,14]. Although a causative relationship is typically inferred, the nature of this relationship is rarely discussed explicitly (e.g. [15,16]). Some studies assume that motion on the fault deforms the weak, magma reservoir (e.g. Donegal [13]), while others show that large batholiths are composite bodies with the individual plutons sequentially emplaced along a preferential alignment (e.g. Adamello [17]; Ardnamurchan [18]). A good illustration of this contrast is given by the Mono Creek Pluton (MCP) and Tuolumne Intrusive Suite (TIS) of the Sierra Nevada batholith (figure 1): the MCP is texturally homogeneous and the internal fabrics are consistent with syn-magmatic NNW-SSE shear, while the TIS consists of a series of at least four compositionally distinct, nested plutons each of which is internally homogeneous and elongated NNW-SSE. These hypotheses are not mutually exclusive but raise an interesting question regarding the nature of the tectonic control: do faults simply act as pathways for magma ascent, or is active deformation important?

Plutons are typically longer than they are thick, with small plutons growing laterally and then inflating vertically [20–22]. In plan view, small plutons tend to be circular and form quickly, while larger ones are more elongated and grow over longer time periods [19]. De Saint Blanquat *et al.* [19] interpret this in the context of active faulting: individual injection events occur sufficiently rapidly that tectonic strain is negligible, whereas the assembly of large plutonic bodies occurs over time scales such that regional tectonic stress fields have the time to deform the growing plutonic body. However, the same observation could be explained if plutonic bodies grow initially by the formation of small magma chambers along a fault, which coalesce into an elongated reservoir that only grows vertically once the surrounding crust is sufficiently hot for ductile deformation. This concept is consistent with the model of Karlstrom *et al.* [22], which shows that the distribution of size preserved intrusions can be approximated by a reverse-energy cascade model of the merging of small intrusions until irreversible deformation and solidification occur.

The orientation of calderas can be used as a proxy for the shape of the magma reservoir, and the orientation of elliptical calderas can therefore be used to distinguish between the influence of active (e.g. [23]) and pre-existing structures (e.g. [24]). In the East African Rift, for example, many calderas are aligned with the major, pre-existing, cross-cutting faults rather than the current stress field [25,26], suggesting that in this case at least, passive pathways are as important as active strain.

### (b) Geophysical evidence

Pressure changes within magma reservoirs can be inferred from surface deformation. Over 220 volcanoes are known to have deformed in the last few decades and while some examples can be attributed to hydrothermal or post-eruption activity, yet more have been linked to magmatic processes [27]. To the first order, the classic ‘volcano deformation cycle' pattern of radially symmetric co-eruptive subsidence and inter-eruptive uplift has been observed at a number of volcanoes with different characteristic lengthscales and time scales [27]. Yet, a recent global review showed that while deformation has a strong statistical link to eruption, only half of all volcanoes that exhibited deformation erupted [28].

**Figure 2.**
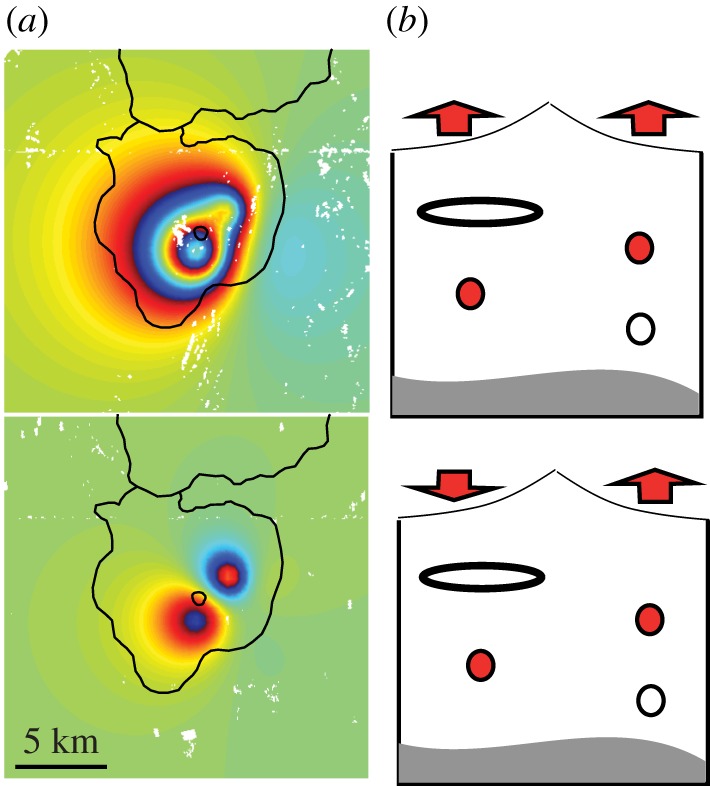
(*a*) Modelled surface deformation at Paka Volcano. Kenya showing multiple sources of pressurization, consistent with multiple aligned magma chambers. Each coloured fringe corresponds to 2.8 cm of deformation in the satellite line of sight. Circle shows the vent and the black line is the extent of the edifice (with Silali to the north). (*b*) Schematic E-W cross-section showing approximate location and depth of modelled sources. After [29]. (Online version in colour.)

High-resolution maps of volcano deformation reveal complexity that was not apparent during the era when volcano geodesy was based on infrequent point measurements (figure 2). The simplest model, that of a point source in an elastic half-space [30], satisfies the observations in a surprisingly large number of cases, but multiple sources, offsets from the vent or time-varying pressure histories are often required (e.g. [31–34]. In other cases, the deformation is asymmetrical and requires complex source geometries, such as ellipsoidal magma bodies or elongated sills (e.g. [35,36]). These observations are consistent with the concept of a magma reservoir composed of a crystalline mush containing multiple lenses of high melt-fraction magma. The time is right to consider the conditions of magma storage, particularly the thermo-mechanical history of the reservoir, when interpreting geodetic signals.

Large regions (tens of km^3^) of partial melt (greater than 10%) have been identified at depths of 5–20 km using geophysical methods including seismology, magnetotellurics and gravity (e.g. [37,38]) and references therein). Many of these are asymmetrical with respect to the surface volcanic features, or even offset (e.g. [39–41]). Several mechanisms are consistent with the observation that zones of partial melt are aligned with fault systems (e.g. [42]): (i) the fault zone acts as a pathway for magma ascent, (ii) the magma is accommodated by motion on the fault zone forming a local patch of extension, and (iii) the shape results from the shearing of a weak magma body.

### (c) Surface manifestations

Magma, hydrothermal fluids and volcanic gases have low density and viscosity and it is likely that they rise almost vertically, exploiting the most convenient pathway with minimum lateral transport (except in a few cases where there is an impermeable cap near the surface). Thus, the patterns of vents, fumaroles and degassing measured at the surface reflect the lateral extent of the underlying magmatic reservoir [43].

Radial and tangential patterns in the distribution of cones and vents [44,45] or in subsurface intrusions [46–48] can be attributed to the local stress field, which is a superposition of stresses from regional tectonics, topographic load and magma pressure (e.g. [45,49,50]). Caldera systems are typically low relief, so the confining stress caused by the topographic load is small, but cycles of uplift and subsidence are commonly observed (e.g. [51,52]) associated with a radially symmetric but temporally variable stress field [53].

However, in many cases, alignments of cones and vents are associated with shallow faults, which can be attributed to the active regional stress regime (e.g. [54]), inherited basement structures (e.g. [55]) or caldera formation (e.g. [56,57]). Distinction between active and pre-existing faults is complicated by the tendency for older faults to be reactivated by active stress fields (e.g. [58,59]).

### (d) Outline

**Figure 3.**
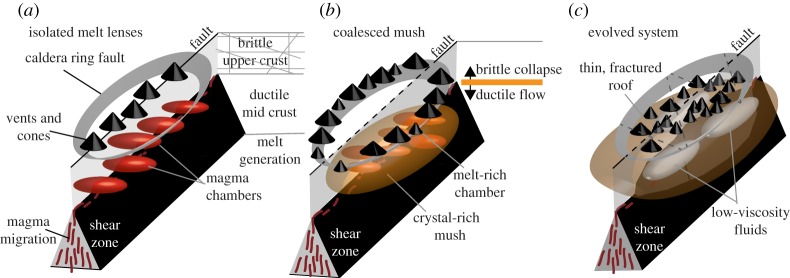
Conceptual model of the growth of magma reservoirs from (*a*) isolated melt lenses aligned along a fault to (*b*) mature reservoirs whose surface characteristics are controlled by a caldera ring fault and finally (*c*) highly evolved mush systems which undergo frequent internal reorganization. (Online version in colour.)

Based on the previous review, we propose a conceptual model of magma reservoir development based on the principle that magma reservoirs grow through short, discrete injections, starting with small, transient magma chambers aligned with faults, which coalesce over time to produce an elongate region of partial melt. According to this conceptual model, the thermal evolution of the reservoir can be subdivided into three distinct phases, each associated with a different reservoir size, shape, gas content and rheology (figure 3). The difference in lateral extent of the magma reservoir mean fluids in the crust above the reservoir would exploit different pathways, causing observable differences in the distribution of volcanic vents, fumaroles and degassing. We identify and discuss modern analogues for each of these stages.

We then consider the response of the magma system to a fresh input of magma, and conclude that the expected patterns of surface deformation would be distinct. Thus we show that (a) the patterns of surface deformation can be used to infer some aspects of the thermal history of the reservoir and (b) the thermal history of the reservoir must be considered when interpreting patterns of surface deformation. Finally, we develop simple thermal models to investigate the time scales over which closely spaced magma bodies would coalesce, with particular reference to (a) the viscoelastic aureole and (b) the region of partial melt. These thermal models do not involve mechanical processes and thus offer upper bounds on the time scales involved.

## 2. Conceptual model

### (a) Stage 1: aligned melt lenses

Discrete batches of magma rise upward along mid-crustal shear zones forming a line of small magma chambers at rheological or lithological boundaries (figure 3*a*). Each magma chamber produces individual deformation patterns, which may overlap in space and time. The magmatic and hydrothermal systems are localized above the shear zone, such that magmatic and hydrothermal fluids exploit the shallow extension of the fault as they rise to the surface. Fumaroles and volcanic vents, many of them monogenetic, are thus aligned along the dominant structure.

Examples include *Puyehue Cordon-Caulle, Chile,* which lies at the intersection of the major trench-parallel strike-slip system, the Liquiñe-Ofqui Fault Zone, (LOFZ) and an old basement structure [55]. Deformation associated with the 2011 eruption indicates the involvement of multiple small magma bodies aligned along this inherited structure [33,60]. Another example, *Corbetti, Ethiopia*, lies at the intersection of the Main Ethiopian Rift with the Goba–Bonga lineament, which has had a major influence on the tectonic evolution of the rift system [61,62]. Evidence for the influence of the cross-rift structure on the magmatic system comes from a number of directions and a range of time scales: patterns of surface deformation are semicircular, bounded by the fault; seismicity is aligned along the fault, and the distribution of volcanic vents is statistically closer to the line than a random distribution [26]. In both these cases, there is also evidence that a larger magma reservoir is active during different time periods [63–65], suggesting a transition in behaviour towards stage 2.

### (b) Stage 2: mature reservoir

The individual chambers coalesce into a large, elongated reservoir containing many small chambers (figure 3*b*). The large magmatic and hydrothermal systems extend beneath the caldera ring fault, which is exploited as a pathway for rising fluids. Coalescence can take place through a number of mechanisms depending on depth and geometry, for example the brittle collapse of isolated blocks of wall-rock or ductile flow. The deformation pattern is associated with the viscoelastic behaviour of the reservoir, which may be elongated in shape. The behaviour of the individual chambers may be reflected in the time-dependent pressure history. The magmatic and hydrothermal systems are now laterally extensive and the dominant pathway for magmatic and hydrothermal fluids is the caldera ring fault. The result is a ring-shaped edifice, with volcanic vents and fumaroles distributed around the caldera rim.

Examples include *Laguna del Maule, Chile*, *Long Valley, USA*, and *Aluto, Ethiopia*, which demonstrate that the presence of a ring fault controls the surface expression of magmatic systems regardless of their tectonic setting. At *Laguna del Maule*, postglacial silicic eruptions form a concentric ring around a central lake and in recent years there has been a prolonged pulse of rapid uplift [57,66]. At *Long Valley*, recent eruptions are concentrated along the caldera and regional fault systems [67] with seismicity along the southern caldera fault [68]. By contrast, multiyear inflation episodes in 1990–1995 and 2011–2014 were driven by a source in the centre of the caldera [69]. At *Aluto, Ethiopia,* the caldera ring fault acts as the major pathway for degassing, hydrothermal upwelling and magmatic fluids [56,70,71]. Aluto has been subsiding for many years, with occasional pulses of rapid uplift [72,73].

### (c) Stage 3: evolved system

Eventually, the large reservoir reorganizes as low-viscosity fluid rise through the crystal-rich mush. Faults and fractures within the extended roof are reactivated and magmatic and hydrothermal fluids exploit these pathways forming volcanic vents and fumaroles distributed across the caldera floor (figure 3*c*). Owing to the large size of the reservoir and the ability of mush to reorganize internally, fresh magma input does not necessarily result in surface deformation. However, frequent, spatially complex deformation is caused by migration of fluid between sources and phase changes with the mush. Numerous cycles of deformation reactivate local and regional faults and fractures across the highly extended caldera floor. Fluids from the laterally extensive hydrothermal and magmatic systems rise along these pathways leaving volcanic vents and fumaroles distributed across the caldera floor.

The classic example is *Yellowstone, USA*, which is an evolved magmatic system with frequent deformation, active hydrothermal system and eruptions from vents distributed across the caldera floor [74–76]. Seismic tomography shows that the shallowest portion of the crustal magma reservoir is overlain by a highly fractured, fluid-filled region, suggesting that magmatic fluids (gas, hydrothermal fluids and melt) have migrated to shallower depths along existing fractures [77]. *Campi Flegrei, Italy*, is an interesting example as it appears to lie at the border between stages 2 and 3. It has experienced multiple pulses of uplift which are attributed to interactions between magmatic and hydrothermal system [78]. Older vents (12–8 kyr) are distributed along the marginal faults of the Neapolitan Yellow Tuff caldera, while younger vents (less than 5 kyr) occur on the caldera floor, which is dissected by reactivation of a number of regional fault systems [79].

### (d) Associated deformation

Surface deformation and eruption occur in response to pressure changes within a magma reservoir; thus to understand deformation patterns and the types of precursory activity that might precede eruptions, it is first necessary to understand what causes and controls changes in pressure within the magma reservoir. In the first case, we consider the input of new magma into a spherical reservoir of volume V at a mass injection rate, d*M*/d*t.* This causes a viscoelastic response in the crust, plus changes in pressure d*P*/d*t,* temperature d*T*/d*t* and gas fraction d*ε_γ_*/d*t* [80–83]. Processes of crystallization and exsolution further alter the relative abundance of crystal, melt and gas phases, such that the mixture density evolves with time; for example, significant overpressure can also be generated by ‘second boiling' [84]. To fully describe the thermo-mechanical evolution of the reservoir requires coupled equations for the conservation of mass, water and energy, but here we illustrate the dominant controls on reservoir pressure using the conservation of mass following fresh magma input (following [83]),

2.1 $$\frac{\text{d}M}{\text{d}t}=\rho V\left[\frac{\Delta P}{\eta_{r}}+\left(\frac{1}{\beta_{m}}+\frac{1}{\beta_{r}}\right)\frac{\text{d}P}{\text{d}t}+\alpha^{*}\frac{\text{d}T}{\text{d}t}+\rho^{*}\frac{\text{d}\varepsilon_{g}}{\text{d}t}\right],$$

where *β_m_* and *β_r_* are the compressibility of the magma and reservoir, respectively, the term Δ*P*/*η_r_* describes the viscous growth in response to an overpressure, *ΔP* according to the effective viscosity. *η*_r_ and α* and ρ*ρ* are coefficients associated with thermal expansion and density defined fully in Degruyter & Huber [83].

For a given mass input, the rate of pressurization is determined by the reservoir size, *V*, gas content (through magma compressibility, *β_c_*) and reservoir shape (through reservoir compressibility, *β_r_*) [85]. Degruyter *et al*. [85] only consider spherical chambers, but we note that the compressibility of a spherical cavity is always less than that for an ellipsoidal cavity or penny-shaped crack [86] which leads to a mismatch between chamber and dyke volume for intrusions [87]. Thus we consider reservoir shape, *β_r,_* as a third important control on magma pressurization. Finally, the influence of the viscoelastic shell of changing dimensions can be investigated using the analytical solution for a spherical chamber of radius *R_1_* within a viscoelastic shell radius of *R_2_*, resulting from a pressure change *P* [80,88]. At *t* = 0, the deformation is equivalent to a chamber of radius *R_1_* embedded in a purely elastic medium and at *t *→* *∞, the deformation pattern approaches that of a magma chamber of radius *R_2_*, thus the effective radius of the chamber increases from *R*_1_ to *R*_2_ with time since the pressure change. Increasing the width of the shell with respect to the chamber radius (*R*_2_/*R*_1_) increases both the characteristic decay time and the magnitude of surface displacement [80,88]. The importance of the viscoelastic response likely depends on the balance between the characteristic viscoelastic time scale (e.g. Maxwell) and the magma flow time scale (e.g. Poiseuille) which are in turn controlled by magma and host rock viscosity and depth [89]. However, the Maxwell time scale for crust with a ‘typical' viscosity on the order of 10^18^ Pas is years to decades, compatible with the time scales for geodetically detected volcanic unrest.

This paradigm is most appropriate for describing small, transient reservoirs with a high melt fraction because the mechanisms of multiphase flow operating within an extensive mush, while still poorly understood, are likely to be significantly different. One hypothesis is that magma migrates upwards through a reorganization of melt and mush which does not intrinsically cause any significant volume change [90], and that surface deformation is driven solely by phase changes, particularly gas exsolution and crystallization [9]. However, melt segregation occurs over long time scales (10^3^–10^5^ years) and over the short time scales associated with unrest (10^−1^–10^1^ years) it is feasible to assume that only the melt and gas components are mobile and that the mush behaves as a viscoelastic medium surrounding the chamber.

**Figure 4.**
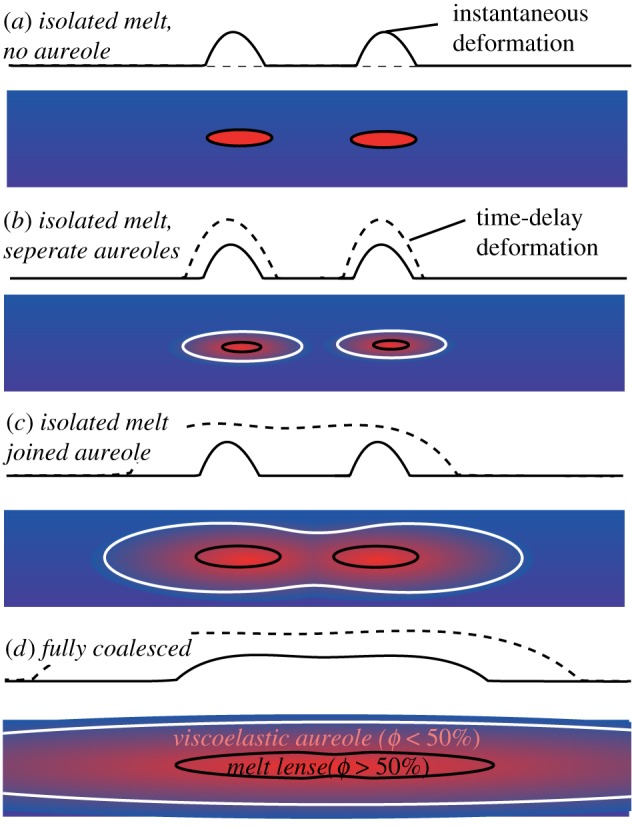
Conceptual model of the coalescence of magma bodies and their viscoelastic aureoles, and the influence on surface deformation. Solid lines show the instantaneous elastic deformation and the dashed lines show the integrated deformation over the longer term, including the viscoelastic response. Numerical models (figure 7) show that stage 4 is rarely achievable at realistic magma fluxes. (Online version in colour.)

Within this framework, the key parameters that determine the response to an intrusion are the size, shape, gas content and thermal aureole of a magma reservoir, which in turn depend on its long-term history of recharge and eruption. From this perspective, the earliest stage of our conceptual model can be further subdivided: (a) where isolated melt bodies intrude cold, elastic crust producing instantaneous deformation, but no viscoelastic response and (b) repeated intrusions have produced a viscoelastic aureole and hence long-term deformation, but these remain spatially independent (figure 4*a,b*). The second stage of the conceptual model corresponds to a situation where the melt bodies remain separated, but the magma reservoirs have coalesced producing a single long-term signal in response to transient pressure changes in one or more melt lens (figure 4*c*). Finally, if the melt supply is large enough to overcome the cooling between intrusions, the melt lenses themselves may coalesce to form a single, large magma chamber (figure 4*d*). Whether or not such a situation is physically plausible is an interesting question, and we will use thermal models to address it in the subsequent sections. However, it seems likely that such a situation would only arise in unusual conditions and not be stable for long periods.

## 3. Numerical models

To fully reproduce the conceptual model described above would require a complex simulation incorporating thermal and mechanical processes within a heterogeneous and fractured crust. Such simulations are beyond the scope of this paper, and would be challenging to interpret in terms of the key processes. Instead, we focus on the temperature field associated with closely spaced intrusions and investigate the time scales over which the viscoelastic aureole and magma reservoir (zone of partial melt) would coalesce through purely thermal processes. This approach implicitly assumes that magma chambers converge when the country rock between intrusions partially melts, and is likely an upper bound on the time scales of coalescence as mechanical links via viscous deformation or hydraulic conduits will be facilitated by increasing temperature and depth.

### (a) Thermal modelling

To construct thermal models of the evolution of magma reservoirs, we adapt the finite-difference numerical scheme described by Annen [5]. Heat transfer is calculated by solving the time-dependent heat equation for conduction in a solid medium (3.1), including the latent heat (*L*) associated with phase changes determined from experimental temperature (*T*) - melt fraction (*X*) relationships; *ρ*, *c_p_*, *L*, *k* are density, specific heat capacity, latent heat and thermal conductivity.

3.1 $$\rho c_{p}\frac{\partial T}{\partial t}+\rho L\frac{\partial X}{\partial t}=k\nabla^{2}T.$$

**Figure 5.**
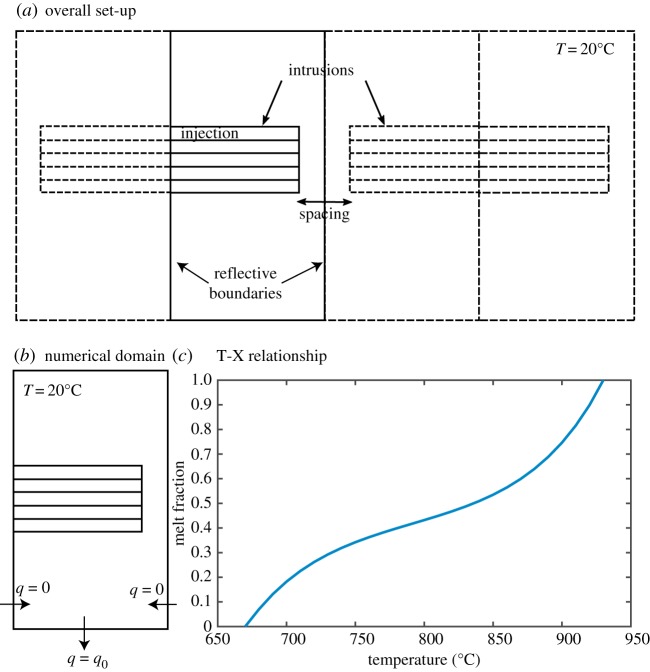
Set-up of the numerical simulations. The numerical domain is outlined with a solid line. The intrusions grow from roof downwards by stacking of sill-like injections. The presence of several intrusions is accounted by imposing a reflective boundary at the right border of the numerical domain. (*b*) The numerical domain showing boundary conditions. (*c*)The relationship between melt fractions and temperatures used in the numerical simulation from Caricchi & Blundy [91]. (Online version in colour.)

The intrusions, reservoir and surrounding country rock are discretized and the temperature and melt fraction of each cell tracked. The intrusions grow by the accretion of successive sill-like injections (figure 5). Rather than specifying an axisymmetric geometry and using a two-dimensional radial slice as in Annen [5], we use Cartesian coordinates with a reflective boundary condition (figure 5). This means the lateral heat flux out of the system is matched by an incoming flux to simulate the effect of a neighbouring magma body. The disadvantage of this geometry is that it assumes the body is infinitely long in the third dimension and thus does not capture the full three-dimensional shape of the reservoir and hence overestimates the rate of heat transport. Nonetheless, the computational advantages of using a simple geometry enable us to explore the parameter space more fully and investigate the first-order controls and time scales over which melt bodies and their surrounding aureoles coalesce.

To explore the range of possible chamber shapes and conditions, it is necessary to explore the parameter space defined by (i) the time-averaged growth rate of the intrusion, (ii) the depth of the intrusions, (iii) the spacing between intrusions. The vertical growth rate of the intrusions is determined by the injection thicknesses and the frequency of injections. Cells with crystal fractions less than 0.5 are considered part of the chamber, and those with temperatures greater than the background geotherm as part of the viscoelastic shell. While ideal for characterising stage 1 of the conceptual model, care must be taken when applying this approach to the dynamics of mush systems, which dominate during stage 3, and operate to some extent, during stage 2.

In this preliminary study, we explore growth rates of 0.1, 0.05 and 0.025 m per year, corresponding, respectively, to injections of 200 m thick emplaced every 2000, 4000 and 8000 years over a total duration of 50 000, 100 000, 200 000 years. At lower growth rates, the magma solidifies between injections and no large magma chamber is generated [92]. Whether higher growth rates are realistic is a debated issue. Regardless of this issue, results tend to plateau at high growth rates and the system behaves as if the whole body of magma was instantaneously emplaced [93]. The exact injection thickness does not affect significantly the results, provided the growth rate is kept constant by adjusting the frequency. After the intrusion has reached a cumulated thickness of 5 km, the system is left to thermally relax during a period of time equivalent to the growth duration. We tested intrusions growing downwards from 5 to 10 km depth, 8 to 13 km depth and 12 to 17 km depth. The horizontal spacing between two intrusions is 0.5, 1 or 2 km. The parameters used in the simulation are reported in table 1. The same properties are used for the intrusion and the country rock. The relationship between melt fraction, *X*, and temperature, *T*, is from Caricchi & Blundy [91] and is shown in figure 5:

3.2 $$\begin{array}{c} X=1-(a+b.*\text{sin}h(c.*T^{\prime }+d)) \end{array}$$

3.3 $$\begin{array}{c} \text{with}\;T^{\prime }=\frac{T-T_{s}}{T_{l}-T_{s}}, \end{array}$$

*T*_*s*_ is the solidus at 670°C, *T*_*l*_ is the liquidus at 930°C, *a* = 5.8213 × 10^−1^, *b* = 8.9078 × 10^−2^, *c* = −4.8306 and *d* = 2.257.

The viscosity prior to melting is given by

3.4 $$\eta =A_{d}\exp\left(\frac{H}{RT}\right),$$

*A_d_*, the Dorn parameter, is 10^9^ Pa s [94,95], *H*, the activation energy of creep mechanism, is 135 000 J mol^−1^ [96] and *R* is the perfect gas constant. The viscosity is only calculated in the absence of melt as equation (3.4) is not valid above solidus.

### (b) Results

We calculated temperatures, viscosities and melt fractions mid-way between two intrusions, i.e. on the right reflective boundary of the numerical domain (figure 5). We expect the heat flux at this point to be twice that from an individual intrusion at a similar distance. We recorded the time needed for the first melt to appear at this boundary, corresponding to the coalescence of the mush.

**Figure 6.**
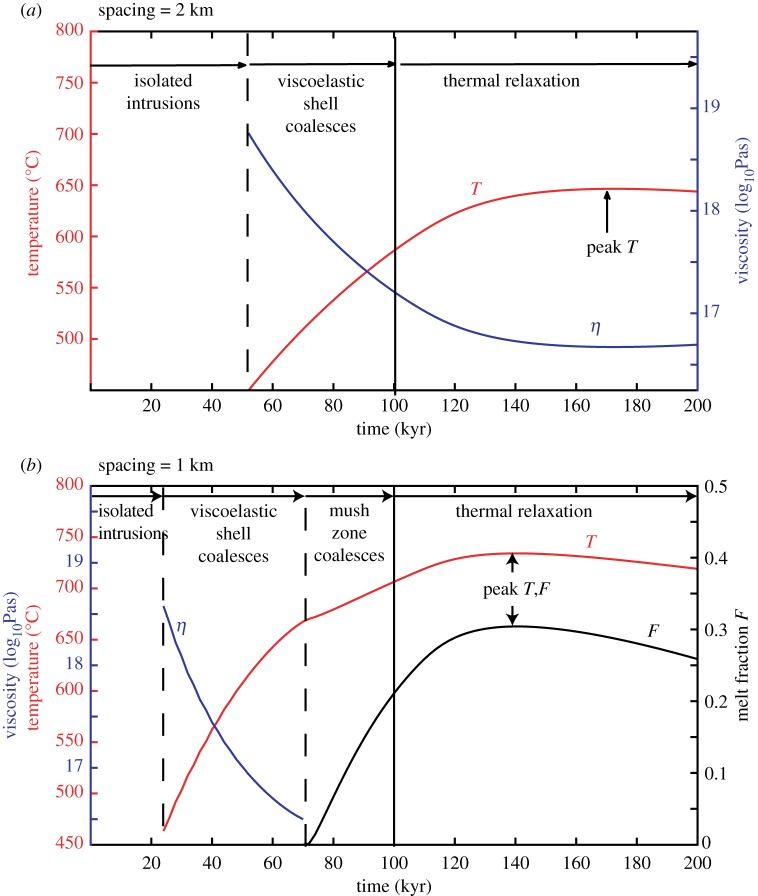
Example of simulation for intrusions growing from 8 to 13 km depth over 100 kyr showing the evolution of the peak temperature, viscosity and melt fraction mid-way between intrusions. The dashed lines mark the first change in temperature and the appearance of melt and the plain line the last magma injection. The viscosity computation is only valid before melt appearance. (*a*) For a spacing of 2 km in which no melt appears, and (*b*) for a spacing of 1 km, where melt appears on the boundary approximately 70 kyr after the start of injection. (Online version in colour.)

Figure 6 illustrates the evolution of the maximum temperatures and viscosities mid-way between intrusions, for example with spacings of 2 km (figure 6*a*) and 1 km (figure 6*b*). In both the cases shown, the intrusions grow downwards from 8 to 15 km depth over 100 kyr. For a spacing of 2 km, the temperature on the reflecting boundary starts to increase after approximately 50 kyr, but does not reach high enough temperatures for melt to form. The viscosity gradually drops from 10^19^ Pas, reaching 10^17^ Pas after 120 kyr forming a single viscoelastic shell surrounding two distinct mush systems (figure 6*a*). For a spacing of 1 km, the temperature starts to increase 18 kyr after the first magma injections, and the viscosity drops to 10^16^ Pas before the first melt appears on the reflective boundary 71 kyr after the first injection. The temperature and melt fraction peak at 140 kyr, equivalent to 40 kyr after the last injection. The maximum melt fraction is 0.3, which means although the mush system has coalesced, the eruptible melt lenses remain isolated (figure 6*b*).

**Figure 7.**
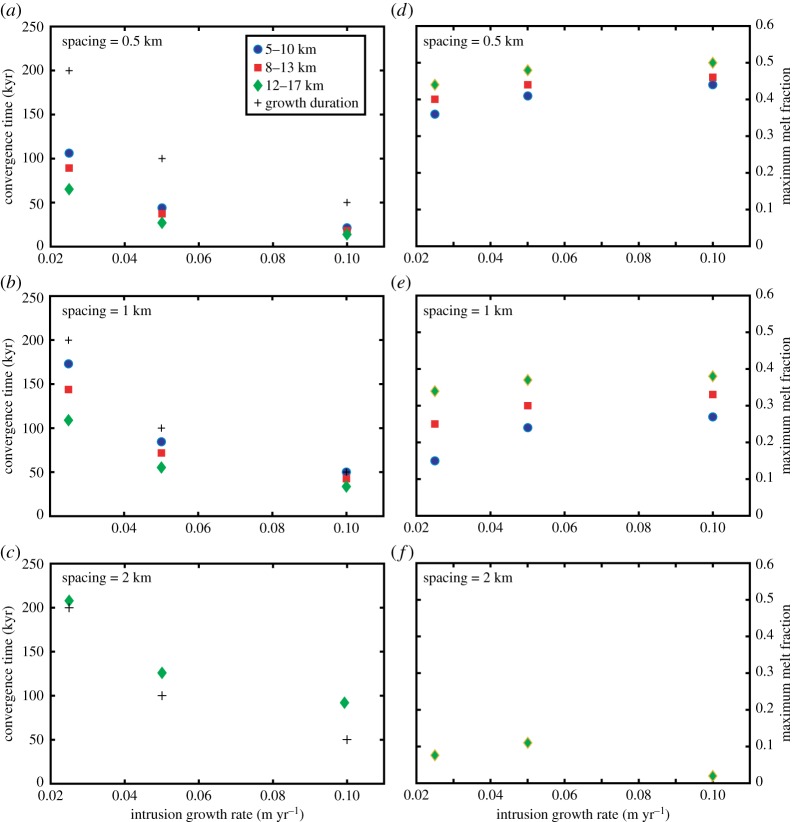
(*a*–*c*) Time elapsed between the first injection and mush convergence as a function of intrusion growth rate and emplacement depth. Spacing between intrusions is 0.5 km (*a*), 1 km (*b*) and 2 km (*c*). For an intrusion spacing of 2 km, convergence is obtained only for intrusions growing from 12 to 17 km. For spacing of 0.5 and 1 km, coalescence occurs during the intrusion growth, whereas for a spacing of 2 km, it occurs after the last magma injection. (*d*–*f*) Maximum melt fraction mid-way between intrusions as a function of intrusion growth rate and emplacement depth. Spacing between intrusions is 0.5 km (*d*), 1 km (*e*) and 2 km (*f*). For an intrusion spacing of 2 km, melt is present only for intrusions growing from 12 to 17 km. (Online version in colour.)

We then compare simulations for a range of magma fluxes, intrusion spacings and depth. As expected, the maximum temperature and melt fraction between the intrusions increases with depth and magma injection rates and hence the time taken for coalescence also decreases (figure 7). Furthermore, when the spacing between the two intrusions decreases, the maximum temperature and melt fraction increase and the time to mush coalescence decreases. This is primarily because the distance between the intrusions and the midway point has decreased. For the largest spacing tested (2 km), the mush zones only coalesce for the deepest experiment (12–17 km), and in this case, coalescence takes place as the system thermally relaxes, after the end of magma injections (figure 7).

**Figure 8.**
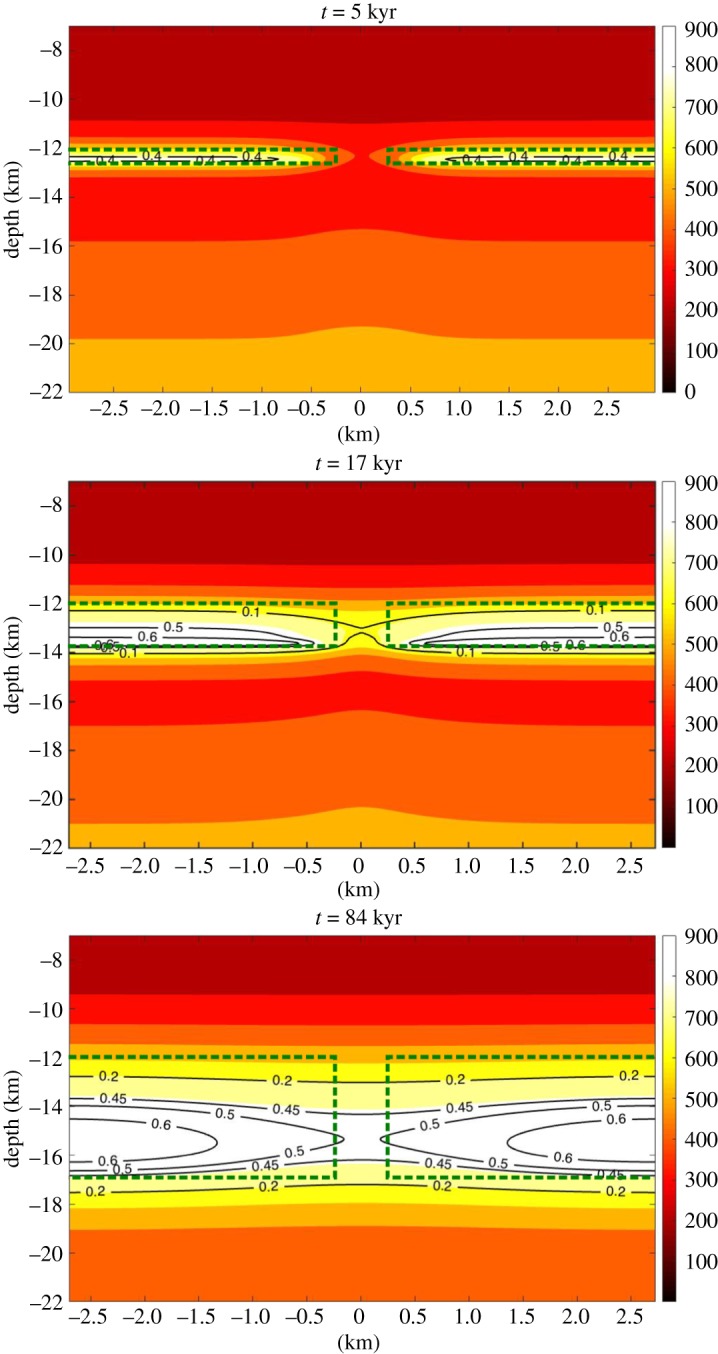
Snapshots at 5 kyr, 17 kyr and 84 kyr after the first magma injection. The intrusions grow downwards from 12 k to 17 km depth over 50 kyr. The spacing between intrusion is 0.5 km. The green dashed lines mark the contour of the intrusions. The temperatures are shown by the colour scale and the melt fraction with the contour lines. At 5 kyr, the intrusions are independent with separate thermal aureoles. At 17 kyr, the mushes converge. At 84 kyr, 34 ky after the last injection, the magma chambers converge. (Online version in colour.)

The convergence of magma chambers (melt fraction ≅0.5) is reached in only one of our simulations—at the closest spacing (0.5 km) and greatest depths (12–17 km) tested. Snapshots of this simulation are shown in figure 8. After 17 kyr, the viscoelastic aureoles have coalesced to produce a single mush system, but the melt bodies remain separated. After 84 kyr, the melt fraction between intrusion is 0.49 and the melt bodies are coalescing into one large magma chamber. Interestingly, this happens 34 kyr after the intrusion has ceased growing.

## 4. Discussion

The preliminary simulations presented here confirm that our conceptual model of the coalescence between magmatic bodies is plausible. A brief exploration of the physically realistic parameter space suggests that extensive low melt-fraction mush systems can develop over the time scales typical for magma injection, but coalescences of melt-rich lenses is rare if due only to conductive heating and partial melting. An interesting result is that, due to slow heat diffusion, coalescence may happen after the main intrusive episodes, so that major caldera eruptions do not necessary coincide in time with the main intrusive activity. The calculations are limited by the fact that they were two dimensional and did not involve any mechanical processes; no convective processes or fluid migration was considered and we tested only for one type of rock composition and melting behaviour. Nevertheless, our calculations suggest these models have the potential to explain many of the observed features of large magma reservoirs and deserve further exploration.

Within this framework, mushes coalesce and magma chambers converge, when the country rock between intrusions partially melts. The ability of rocks to melt strongly depends on their composition and H_2_O content. In the cases presented here, compositions are dacitic and melt appears at 670°C. Moreover, the melting behaviour of injected magma and country rock is similar. In nature, if the country rocks is dehydrated and refractory, mush coalescence would not happen. By contrast, coalescence would be favoured if a hot mafic magma is injected in a wet fertile country rock [97].

For most of the parameter space explored, the melt fraction between the intrusions remained below 0.4, although the wider mush system had coalesced. In a real system, we might expect the size of the magma chambers to be reduced by eruption of mobile, high melt fraction material, while the surrounding immobile mush continues to grow. This suggests individual melt lenses can remain isolated for long periods within growing mush systems, and will only mix during eruptions or other catastrophic events.

In our numerical models, for the melt lenses to coalesce, they must be deep, close to each other, and the magma supply rate must be sufficiently high. In nature, a physical disruption of the mush between the magma chambers could occur at low melt fractions and lead to a merging of the chambers. This disruption can be caused by a tectonic event or by an eruption from one of the chambers and the ensuing magma depressurization (e.g. [98]). The connection of formerly separated melt lenses during caldera forming eruptions has been identified from the petrological and geochemical record of the erupted products [99–101], and from geophysical observations (e.g. [33,102]).

Finally, although these models do not directly assess the structural controls of magma reservoir growth, they clearly demonstrate that mush zones preferentially develop between closely spaced intrusions. Thus growth will be favoured in directions along which structural features cause intrusions to align, and inhibited away from them. Possible mechanisms for such an alignment might include an increase in permeability, stress focusing or active fault motion, but this study does not discriminate between them.

## 5. Conclusion

We have presented a new conceptual model that integrates short-term observations of volcanic systems, with the long-term growth of magmatic systems. We adopt the premise that systems start as a set of closely spaced, but isolated intrusions which grow and coalesce through thermal diffusion. We identify three distinct stages of coalescence: the viscoelastic shell, the solidus (melt fraction greater than 0) and the eruptible melt (melt fraction greater than 0.5). We explore a two-dimensional numerical model using a reflective boundary condition to simulate the growth of closely spaced reservoirs and track the conditions midway between intrusions. In most simulations, the temperature starts to rise after 10s kyr, indicating that the viscoelastic aureole has coalesced, and the melt fraction starts to rise after 10–100s kyr. The temperature and melt fraction are greatest for deep, closely spaced intrusions at high magma supply rates, and coalescence occurs most rapidly in these conditions. The temperature and melt fraction peak during a period of thermal relaxation suggesting the optimal conditions for caldera-forming eruptions might occur 10s kyr after the magma supply has ceased. For most of the simulations, melt fraction does not reach 0.5, indicating that individual lenses of eruptible magma remain isolated within a larger mush system.

## Data accessibility

The code used for the thermal modelling is deposited on runmycode.org.

## References

[1] Brown M 1994 The generation, segregation, ascent and emplacement of granite magma: the migmatite-to-crustally-derived granite connection in thickened orogens. Earth Sci. Rev. 36, 83–130. (10.1016/0012-8252(94)90009-4)

[2] Brown M 2013 Granite: From genesis to emplacement. Geol. Soc. Am. Bull. 125, 1079–1113. (10.1130/B30877.1)

[3] Gudmundsson A 2011 Deflection of dykes into sills at discontinuities and magma-chamber formation. Tectonophysics 500, 50–64. (10.1016/j.tecto.2009.10.015)

[4] Menand T 2008 The mechanics and dynamics of sills in layered elastic rocks and their implications for the growth of laccoliths and other igneous complexes. Earth Planet. Sci. Lett. 267, 93–99. (10.1016/j.epsl.2007.11.043)

[5] Annen C 2009 From plutons to magma chambers: thermal constraints on the accumulation of eruptible silicic magma in the upper crust. Earth Planet. Sci. Lett. 284, 409–416. (10.1016/j.epsl.2009.05.006)

[6] de Silva SL, Gosnold WD 2007 Episodic construction of batholiths: insights from the spatiotemporal development of an ignimbrite flare-up. J. Volcanol. Geotherm. Res. 167, 320–335. (10.1016/j.jvolgeores.2007.07.015)

[7] Glazner AF, Bartley JM, Coleman DS, Gray W, Taylor RZ 2004 Are plutons assembled over millions of years by amalgamation from small magma chambers? GSA today 14, 4–12. (10.1130/1052-5173(2004)014%3C0004:APAOMO%3E2.0.CO;2)

[8] Bachmann O, Bergantz G 2008 The magma reservoirs that feed supereruptions. Elements 4, 17–21. (10.2113/GSELEMENTS.4.1.17)

[9] Sparks RSJ, Cashman KV 2017 Dynamic magma systems: implications for forecasting volcanic activity. Elements 13, 35–40. (10.2113/gselements.13.1.35)

[10] Bachmann O, Bergantz GW 2004 On the origin of crystal-poor rhyolites: extracted from batholithic crystal mushes. J. Petrol. 45, 1565–1582. (10.1093/petrology/egh019)

[11] Cashman KV, Giordano G 2014 Calderas and magma reservoirs. J. Volcanol. Geotherm. Res. 288, 28–45. (10.1016/j.jvolgeores.2014.09.007)

[12] Parmigiani A, Faroughi S, Huber C, Bachmann O, Su Y 2016 Bubble accumulation and its role in the evolution of magma reservoirs in the upper crust. Nature 532, 492–495. (10.1038/nature17401) 27074507

[13] Hutton D 1982 A tectonic model for the emplacement of the Main Donegal Granite, NW Ireland. J. Geol. Soc. 139, 615–631. (10.1144/gsjgs.139.5.0615)

[14] Hutton DH 1997 Syntectonic granites and the principle of effective stress: A general solution to the space problem? In Granite: from segregation of melt to emplacement fabrics, pp. 189–197. Berlin, Germany: Springer.

[15] D'lemos R, Brown M, Strachan R 1992 Granite magma generation, ascent and emplacement within a transpressional orogen. J. Geol. Soc. 149, 487–490. (10.1144/gsjgs.149.4.0487)

[16] Hutton DH 1988 Granite emplacement mechanisms and tectonic controls: inferences from deformation studies. Trans. R. Soc. Edinburgh Earth Sci. 79, 245–255. (10.1017/S0263593300014255)

[17] Del Moro A, Pardini G, Quercioli C, Villa I, Callegari E 1983 Rb/Sr and K/Ar chronology of Adamello granitoids, southern Alps. Mem. Soc. Geol. Ital 26, e299.

[18] Richey JE, Thomas HH, Radley EG, Dixon B 1930 The geology of Ardnamurchan, north-west Mull and Coll: a description of sheet 51 and part of sheet 52 of the geological map. London, UK: HM Stationery Office.

[19] de Saint Blanquat M, Horsman E, Habert G, Morgan S, Vanderhaeghe O, Law R, Tikoff B 2011 Multiscale magmatic cyclicity, duration of pluton construction, and the paradoxical relationship between tectonism and plutonism in continental arcs. Tectonophysics 500, 20–33. (10.1016/j.tecto.2009.12.009)

[20] McCaffrey K, Petford N 1997 Are granitic intrusions scale invariant? J. Geol. Soc. 154, 1–4. (10.1144/gsjgs.154.1.0001)

[21] Petford N, Cruden A, McCaffrey K, Vigneresse J-L 2000 Granite magma formation, transport and emplacement in the Earth's crust. Nature 408, 669–673. (10.1038/35047000) 11130061

[22] Karlstrom L, Paterson SR, Jellinek AM 2017 A reverse energy cascade for crustal magma transport. Nat. Geosci. 10, 604 (10.1038/ngeo2982)

[23] Bosworth W, Burke K, Strecker M 2003 Effect of stress fields on magma chamber stability and the formation of collapse calderas. Tectonics 22, 1042 (10.1029/2002TC001369)

[24] Acocella V 2003 Elliptic calderas in the Ethiopian Rift: control of pre-existing structures. J. Volcanol. Geotherm. Res. 119, 189–203. (10.1016/S0377-0273(02)00342-6)

[25] Robertson E, Biggs J, Cashman K, Floyd M, Vye-Brown C 2015 Influence of regional tectonics and pre-existing structures on the formation of elliptical calderas in the Kenyan Rift. Geol. Soc. Lond. Spec. Pub. 420, 43–67. (10.1144/SP420.12)

[26] Lloyd R, Biggs J, Wilks M, Nowacki A, Kendall JM, Ayele A, Lewi E, Eysteinsson H 2018 Evidence for cross rift structural controls on deformation and seismicity at a continental rift caldera. Earth Planet. Sci. Lett. 487, 190–200. (10.1016/j.epsl.2018.01.037)

[27] Biggs J, Pritchard ME 2017 Global volcano monitoring: what does it mean when volcanoes deform? Elements 13, 17–22. (10.2113/gselements.13.1.17)

[28] Biggs J, Ebmeier S, Aspinall W, Lu Z, Pritchard M, Sparks R, Mather T 2014 Global link between deformation and volcanic eruption quantified by satellite imagery. Nat. Commun. 5, 3471. 24699342 10.1038/ncomms4471 PMC4409635

[29] Biggs J, Robertson E, Cashman K 2016 The lateral extent of volcanic interactions during unrest and eruption. Nat. Geosci. 9, 308.

[30] Mogi K 1958 Relations between the eruptions of various volcanoes and the deformations of the ground surfaces around them. Bull. Earthquake Res. Inst. Tokyo 36, 99–134.

[31] Biggs J, Amelung F, Gourmelen N, Dixon T, Kim S 2009 InSAR observations of 2007 Tanzania rifting episode reveal mixed fault and dyke extension in an immature continental rift. Geophys. J. Int. 179, 549–558. (10.1111/j.1365-246X.2009.04262.x)

[32] Biggs J, Robertson E, Cashman K 2016 The lateral extent of volcanic interactions during unrest and eruption. Nat. Geosci. 9, 308 (10.1038/ngeo2658)

[33] Jay J, Costa F, Pritchard M, Lara L, Singer B, Herrin J 2014 Locating magma reservoirs using InSAR and petrology before and during the 2011–2012 Cordon Caulle silicic eruption. Earth Planet. Sci. Lett. 395, 254–266. (10.1016/j.epsl.2014.03.046)

[34] Parks MM et al. 2015 From quiescence to unrest: 20years of satellite geodetic measurements at Santorini volcano, Greece. J. Geophys. Res. Solid Earth 120, 1309–1328. (10.1002/2014JB011540)

[35] Biggs J, Mothes P, Ruiz M, Amelung F, Dixon TH, Baker S, Hong SH 2010 Stratovolcano growth by co-eruptive intrusion: the 2008 eruption of Tungurahua Ecuador. Geophys. Res. Lett. 37 (10.1029/2010GL044942)

[36] Lundgren P, Samsonov SV, López Velez CM, Ordoñez M 2015 Deep source model for Nevado del Ruiz Volcano, Colombia, constrained by interferometric synthetic aperture radar observations. Geophys. Res. Lett. 42, 4816–4823. (10.1002/2015GL063858)

[37] Pritchard ME, Gregg PM 2016 Geophysical evidence for silicic crustal melt in the continents: where, what kind, and how much? Elements 12, 121–127. (10.2113/gselements.12.2.121)

[38] Pritchard ME et al. 2018 Synthesis: PLUTONS: investigating the relationship between pluton growth and volcanism in the Central Andes. Geosphere 14, 945–982. (10.1130/GES01578.1)

[39] Desissa M, Johnson N, Whaler K, Hautot S, Fisseha S, Dawes G 2013 A mantle magma reservoir beneath an incipient mid-ocean ridge in Afar, Ethiopia. Nat. Geosci. 6, 861–865. (10.1038/ngeo1925)

[40] Díaz D, Heise W, Zamudio F 2015 Three-dimensional resistivity image of the magmatic system beneath Lastarria volcano and evidence for magmatic intrusion in the back arc (northern Chile). Geophys. Res. Lett. 42, 5212–5218. (10.1002/2015GL064426)

[41] Hill GJ, Bibby HM, Ogawa Y, Wallin EL, Bennie SL, Caldwell TG, Keys H, Bertrand EA, Heise W 2015 Structure of the Tongariro Volcanic system: insights from magnetotelluric imaging. Earth Planet. Sci. Lett. 432, 115–125. (10.1016/j.epsl.2015.10.003)

[42] Saxby J, Gottsmann J, Cashman K, Gutiérrez E 2016 Magma storage in a strike-slip caldera. Nat. Commun. 7, 12295 (10.1038/ncomms12295) 27447932 PMC4961867

[43] Karlstrom L, Wright HM, Bacon CR 2015 The effect of pressurized magma chamber growth on melt migration and pre-caldera vent locations through time at Mount Mazama, Crater Lake, Oregon. Earth Planet. Sci. Lett. 212, 209–219. (10.1016/j.epsl.2014.12.001)

[44] van Wyk de Vries B, Merle O 1996 The effect of volcanic constructs on rift fault patterns. Geology 24, 643–646. (10.1130/0091-7613(1996)024%3C0643:TEOVCO%3E2.3.CO;2)

[45] Muirhead JD, Kattenhorn SA, Le Corvec N 2015 Varying styles of magmatic strain accommodation across the East African Rift. Geochem. Geophys. Geosyst. 16, 2775–2795. (10.1002/2015GC005918)

[46] Bagnardi M, Amelung F, Poland MP 2013 A new model for the growth of basaltic shields based on deformation of Fernandina volcano, Galápagos Islands. Earth Planet. Sci. Lett. 377, 358–366. (10.1016/j.epsl.2013.07.016)

[47] Biggs J, Chivers M, Hutchinson MC 2013 Surface deformation and stress interactions during the 2007–2010 sequence of earthquake, dyke intrusion and eruption in northern Tanzania. Geophys. J. Int. 195, 16–26. (10.1093/gji/ggt226)

[48] Xu W, Jónsson S 2014 The 2007–8 volcanic eruption on Jebel at Tair island (Red Sea) observed by satellite radar and optical images. Bull. Volcanol. 76, 1–14.

[49] Pinel V, Jaupart C 2000 The effect of edifice load on magma ascent beneath a volcano. Phil. Trans. R. Soc. Lond. A 358, 1515–1532. (10.1098/rsta.2000.0601)

[50] Wadge G, Biggs J, Lloyd R, Kendall J-M 2016 Historical volcanism and the state of stress in the East African Rift System. Front. Earth Sci. 4, 86 (10.3389/feart.2016.00086)

[51] Biggs J et al. 2014.

[52] Dvorak JJ, Dzurisin D 1997 Volcano geodesy: the search for magma reservoirs and the formation of eruptive vents. Rev. Geophys. 35, 343–384. (10.1029/97RG00070)

[53] Walter TR, Troll VR 2001 Formation of caldera periphery faults: an experimental study. Bull. Volcanol. 63, 191–203. (10.1007/s004450100135)

[54] Robertson E, Biggs J, Edmonds M, Clor L, Fischer TP, Vye-Brown C, Kianji G, Koros W, Kandie R 2016 Diffuse degassing at Longonot volcano, Kenya: Implications for CO_2_ flux in continental rifts. J. Volcanol. Geotherm. Res. 327, 208–222. (10.1016/j.jvolgeores.2016.06.016)

[55] Cembrano J, Lara L 2009 The link between volcanism and tectonics in the southern volcanic zone of the Chilean Andes: a review. Tectonophysics 471, 96–113. (10.1016/j.tecto.2009.02.038)

[56] Hutchison W, Mather TA, Pyle DM, Biggs J, Yirgu G 2015 Structural controls on fluid pathways in an active rift system: a case study of the Aluto volcanic complex. Geosphere 11, 542–562. (10.1130/GES01119.1)

[57] Singer BS et al. 2014 Dynamics of a large, restless, rhyolitic magma system at Laguna del Maule, southern Andes, Chile. GSA Today 24, 4–10. (10.1130/GSATG216A.1)

[58] Holdsworth R, Stewart M, Imber J, Strachan R 2001 The structure and rheological evolution of reactivated continental fault zones: a review and case study. Geol. Soc. Lond. Special Publications 184, 115–137. (10.1144/GSL.SP.2001.184.01.07)

[59] Sibson RH 1985 A note on fault reactivation. J. Struct. Geol. 7, 751–754. (10.1016/0191-8141(85)90150-6)

[60] Wendt A, Tassara A, Báez JC, Basualto D, Lara LE, García F 2017 Possible structural control on the 2011 eruption of Puyehue-Cordón Caulle volcanic complex (southern Chile) determined by InSAR, GPS and seismicity. Geophys. J. Int. 208, 134–147. (10.1093/gji/ggw355)

[61] Bonini M, Corti G, Innocenti F, Manetti P, Mazzarini F, Abebe T, Pecskay Z 2005 Evolution of the Main Ethiopian Rift in the frame of Afar and Kenya rifts propagation. Tectonics 24, TC1007 (10.1029/2004TC001680)

[62] Corti G, Sani F, Agostini S, Philippon M, Sokoutis D, Willingshofer E 2018 Off-axis volcano-tectonic activity during continental rifting: Insights from the transversal Goba-Bonga lineament, Main Ethiopian Rift (East Africa). Tectonophysics 728, 75–91. (10.1016/j.tecto.2018.02.011)

[63] Lloyd R, Biggs J, Birhanu Y, Wilks M, Gottsmann J, Kendall JM, Ayele A, Lewi E, Eysteinsson H 2018 Sustained Uplift at a Continental Rift Caldera. J. Geophys. Res. Solid Earth 123, 5209–5226.

[64] Delgado F, Pritchard ME, Basualto D, Lazo J, Córdova L, Lara LE 2016 Rapid reinflation following the 2011–2012 rhyodacite eruption at Cordón Caulle volcano (Southern Andes) imaged by InSAR: Evidence for magma reservoir refill. Geophys. Res. Lett. 43, 9552–9562. (10.1002/2016GL070066)

[65] Euillades PA, Euillades LD, Blanco MH, Velez ML, Grosse P, Sosa GJ 2017 Co-eruptive subsidence and post-eruptive uplift associated with the 2011–2012 eruption of Puyehue-Cordón Caulle, Chile, revealed by DInSAR. J. Volcanol. Geotherm. Res. 344, 257–269. (10.1016/j.jvolgeores.2017.06.023)

[66] Feigl KL, Le Mével H, Ali ST, Córdova L, Andersen NL, DeMets C, Singer BS 2014 Rapid uplift in Laguna del Maule volcanic field of the Andean Southern Volcanic zone (Chile) 2007–2012. Geophys. J. Int. 196, 885–901. (10.1093/gji/ggt438)

[67] Hildreth W 2004 Volcanological perspectives on Long Valley, Mammoth Mountain, and Mono Craters: several contiguous but discrete systems. J. Volcanol. Geotherm. Res. 136, 169–198. (10.1016/j.jvolgeores.2004.05.019)

[68] Hill DP, Langbein JO, Prejean S 2003 Relations between seismicity and deformation during unrest in Long Valley Caldera, California, from 1995 through 1999. J. Volcanol. Geotherm. Res. 127, 175–193. (10.1016/S0377-0273(03)00169-0)

[69] Montgomery-Brown et al. 2015.

[70] Braddock M, Biggs J, Watson IM, Hutchison W, Pyle DM, Mather TA 2017 Satellite observations of fumarole activity at Aluto volcano, Ethiopia: Implications for geothermal monitoring and volcanic hazard. J. Volcanol. Geotherm. Res. 341, 70–83. (10.1016/j.jvolgeores.2017.05.006)

[71] Nowacki A, Wilks M, Kendall JM, Biggs J, Ayele A 2018 Characterising hydrothermal fluid pathways beneath Aluto volcano, Main Ethiopian Rift, using shear wave splitting. J. Volcanol. Geotherm. Res. 356, 331–341. (10.1016/j.jvolgeores.2018.03.023)

[72] Biggs J, Bastow ID, Keir D, Lewi E 2011 Pulses of deformation reveal frequently recurring shallow magmatic activity beneath the Main Ethiopian Rift. Geochem. Geophys. Geosyst. 12, Q0AB10 (10.1029/2011GC003662)

[73] Hutchison W et al. 2016 Causes of unrest at silicic calderas in the East African Rift: new constraints from InSAR and soil-gas chemistry at Aluto volcano, Ethiopia. Geochemistry, Geophysics. Geosystems 17, 3008–3030.

[74] Chang W-L, Smith RB, Wicks C, Farrell JM, Puskas CM 2007 Accelerated uplift and magmatic intrusion of the Yellowstone caldera, 2004 to 2006. Science 318, 952–956. (10.1126/science.1146842) 17991858

[75] Wicks C, Thatcher W, Dzurisin D 1998 Migration of fluids beneath Yellowstone caldera inferred from satellite radar interferometry. Science 282, 458–462. (10.1126/science.282.5388.458) 9774269

[76] Wicks CW, Thatcher W, Dzurisin D, Svarc J 2006 Uplift, thermal unrest and magma intrusion at Yellowstone caldera. Nature 440, 72–75. (10.1038/nature04507) 16511491

[77] Farrell J, Smith RB, Husen S, Diehl T 2014 Tomography from 26 years of seismicity revealing that the spatial extent of the Yellowstone crustal magma reservoir extends well beyond the Yellowstone caldera. Geophys. Res. Lett. 41, 3068–3073. (10.1002/2014GL059588)

[78] Chiodini G, Caliro S, Cardellini C, Granieri D, Avino R, Baldini A, Donnini M, Minopoli C 2010 Long-term variations of the Campi Flegrei, Italy, volcanic system as revealed by the monitoring of hydrothermal activity. J. Geophys. Res. 115, B03205 (10.1029/2008JB006258)

[79] Di Vito M, Isaia R, Orsi G, Southon J, De Vita S, d'Antonio M, Pappalardo L, Piochi M 1999 Volcanism and deformation since 12 000 years at the Campi Flegrei caldera (Italy). J. Volcanol. Geotherm. Res. 91, 221–246. (10.1016/S0377-0273(99)00037-2)

[80] Dragoni M, Magnanensi C 1989 Displacement and stress produced by a pressurized, spherical magma chamber, surrounded by a viscoelastic shell. Phys. Earth Planet. Inter. 56, 316–328. (10.1016/0031-9201(89)90166-0)

[81] Jellinek AM, DePaolo DJ 2003 A model for the origin of large silicic magma chambers: precursors of caldera-forming eruptions. Bull. Volc. 65, 363–381. (10.1007/s00445-003-0277-y)

[82] Woods AW, Huppert HE 2003 On magma chamber evolution during slow effusive eruptions. J. Geophys. Res. Solid Earth 108, 2403 (10.1029/2002JB002019)

[83] Degruyter W, Huber C 2014 A model for eruption frequency of upper crustal silicic magma chambers. Earth Planet. Sci. Lett. 403, 117–130. (10.1016/j.epsl.2014.06.047)

[84] Tait S, Jaupart C, Vergniolle S 1989 Pressure, gas content and eruption periodicity of a shallow, crystallising magma chamber. Earth Planet. Sci. Lett. 92, 107–123. (10.1016/0012-821X(89)90025-3)

[85] Degruyter W, Huber C, Bachmann O, Cooper KM, Kent AJ 2016 Magma reservoir response to transient recharge events: the case of Santorini volcano (Greece). Geology 44, 23–26. (10.1130/G37333.1)

[86] Amoruso A, Crescentini L 2009 Shape and volume change of pressurized ellipsoidal cavities from deformation and seismic data. J. Geophys. Res. Solid Earth 114, B02210.

[87] Rivalta E, Segall P 2008 Magma compressibility and the missing source for some dike intrusions. Geophys. Res. Lett. 35, L04306 (10.1029/2007GL032521)

[88] Segall P 2010 Earthquake and volcano deformation. Princeton, NJ: Princeton University Press.

[89] Le Mével H, Gregg PM, Feigl KL 2016 Magma injection into a long-lived reservoir to explain geodetically measured uplift: Application to the 2007–2014 unrest episode at Laguna del Maule volcanic field, Chile. J. Geophys. Res. Solid Earth 121, 6092–6108. (10.1002/2016JB013066) 27867790 PMC5101856

[90] Christopher T, Edmonds M, Humphreys M, Herd RA 2010 Volcanic gas emissions from Soufrière Hills Volcano, Montserrat 1995–2009, with implications for mafic magma supply and degassing. Geophys. Res. Lett. 37, L00E04 (10.1029/2009GL041325)

[91] Caricchi L, Blundy J 2015 Experimental petrology of monotonous intermediate magmas. Geol. Soc. Lond. Special Publications 422, 105–130. (10.1144/sp422.9)

[92] Annen C 2011 Implications of incremental emplacement of magma bodies for magma differentiation, thermal aureole dimensions, and plutonism-volcanism relationships. Tectonphysics 500, 3–10. (10.1016/j.tecto.2009.04.010)

[93] Blundy J, Annen C 2016 Crustal magmatic systems from the perspective of heat transfer. Elements 12, 115–120. (10.2113/gselements.12.2.115)

[94] Hickey J, Gottsmann J, Nakamichi H, Iguchi M 2016 Thermomechanical controls on magma supply and volcanic deformation: application to Aira caldera, Japan. Sci. Rep. 6, 32691. 27619897 10.1038/srep32691 PMC5020646

[95] Gregg P, de Silva S, Grosfils E, Parmigiani JP 2012 Catastrophic caldera-forming eruptions: thermomechanics and implications for eruption triggering and maximum caldera dimensions on Earth. J. Volcanol. Geotherm. Res. 241, 1–12. (10.1016/j.jvolgeores.2012.06.009)

[96] Meissner R, Tanner B 1992 Crustal viscosities and seismic velocities. Phys. Earth Planet. Inter. 69, 252–256. (10.1016/0031-9201(92)90143-J)

[97] Annen C, Blundy JD, Sparks RSJ. 2008 The sources of granitic melt in Deep Hot Zones. Earth Environ. Sci. Trans. R. Soc. Edinburgh 97, 297–309. (10.1017/S0263593300001462)

[98] Karlstrom L, Rudolph ML, Manga M 2012 Caldera size modulated by the yield stress within a crystal-rich magma reservoir. Nat. Geosci. 5, 402–405. (10.1038/ngeo1453)

[99] Allan ASR, Wilson CJN, Millet M-A, Wysoczanski RJ 2012 The invisible hand: tectonic triggering and modulation of a rhyolitic supereruption. Geology 40, 563–566. (10.1130/g32969.1)

[100] Ellis BS, Wolff JA 2012 Complex storage of rhyolite in the central Snake River Plain. J. Volcanol. Geotherm. Res. 211, 1–11. (10.1016/j.jvolgeores.2011.10.002)

[101] Gualda GR, Ghiorso M 2013 The Bishop Tuff giant magma body: an alternative to the Standard Model. Contrib. Mineral. Petrol. 166, 755–775. (10.1007/s00410-013-0901-6)

[102] Pagli C, Wright TJ, Ebinger CJ, Yun SH, Cann JR, Barnie T, Ayele A 2012 Shallow axial magma chamber at the slow-spreading Erta Ale Ridge. Nat. Geosci. 5, 284 (10.1038/ngeo1414)

